# The rare report of unicentric Castleman disease with concurrent myasthenia gravis and paraneoplastic pemphigus: a case report with a focused review of the literature

**DOI:** 10.3389/fonc.2025.1717172

**Published:** 2026-01-12

**Authors:** Zeyu Cheng, Wenliang Liu, Yan Hu

**Affiliations:** 1 Department of Thoracic Surgery, The Second Xiangya Hospital, Central South University, Changsha, Hunan, China; 2 Hunan Key Laboratory of Early Diagnosis and Precision Treatment of Lung Cancer, The Second Xiangya Hospital of Central South University, Changsha, China

**Keywords:** Castleman disease, myasthenia gravis, paraneoplastic pemphigus, case report, multidisciplinary collaborative management

## Abstract

**Rationale:**

Castleman disease (CD) is a rare lymphoproliferative disorder that can be associated with autoimmune manifestations. While paraneoplastic pemphigus (PNP) and myasthenia gravis (MG) have each been reported with CD, their simultaneous occurrence is extremely uncommon. Here, we describe a case of mixed-type unicentric Castleman disease (UCD) complicated by both MG and PNP.

**Patient concerns:**

A 49-year-old man presented with fluctuating right eyelid ptosis, consistent with ocular MG. Several months later, he developed progressive oral erosions.

**Diagnoses:**

The patient was diagnosed with ocular myasthenia gravis (MG) based on fluctuating ptosis and a positive neostigmine test. Chest imaging revealed an anterior mediastinal mass initially suspected to be thymoma. Biopsy and immunopathology of the lips and oral mucosa confirmed PNP, with direct immunofluorescence showing intercellular IgG deposition and serological positivity for envoplakin and periplakin antibodies. After stabilization of systemic symptoms, the mediastinal mass was surgically removed, and histopathological examination confirmed mixed-type UCD. To date, only four previous cases of this triad have been reported in the literature, and the present case represents a mixed-type UCD subtype.

**Interventions:**

The patient was treated with pyridostigmine for MG, systemic corticosteroids and supportive therapy for PNP, followed by complete surgical excision of the mediastinal mass once symptoms were controlled.

**Outcomes:**

Postoperatively, corticosteroid therapy was continued, resulting in near-complete resolution of oral lesions. During a 20-month follow-up period, the patient remained free of MG recurrence and showed no new PNP lesions.

**Lessons:**

This case highlights a rare and diagnostically challenging overlap of CD, MG, and PNP. Accurate recognition required integration of histopathology, immunofluorescence, and serological testing, achieved through multidisciplinary collaboration across thoracic surgery, neurology, dermatology, and pathology. Clinicians should remain vigilant for such complex presentations, as early diagnosis and coordinated management are essential to improving outcomes in rare autoimmune-associated CD.

## Introduction

Castleman disease (CD) is a rare chronic lymphoproliferative disorder, also referred to as angiofollicular or giant lymph node hyperplasia, and was first described by Castleman in 1956. Histologically, it is characterized by preserved nodal architecture with prominent follicular and vascular proliferation (1). Although its pathogenesis remains unclear, associations with viral infections such as HIV, HHV-8, EBV, and CMV have been reported. CD is classified based on lymph node involvement into unicentric (UCD) and multicentric (MCD) forms, the latter including HHV-8–associated, POEMS-associated, and idiopathic subtypes (2, 3). Histological variants include hyaline vascular, plasma cell, and mixed types (4). Among lymphoproliferative disorders, pproximately 18% of PNP cases are reported to be CD-related, most often associated with the unicentric hyaline vascular subtype (5). Clinically, PNP typically presents with painful erosions and ulcerations of the oral mucosa and vermilion border, often as the earliest sign, while histopathology demonstrates acantholysis with interface dermatitis (6). Over the past few years, comprehensive reviews and systematic analyses have highlighted that plakin-family autoantibodies, particularly envoplakin and periplakin, are central to the pathogenesis of PNP (7, 8). In addition, recent evidence indicates that effective treatment of the underlying neoplasm leads to clinical improvement in approximately 63.4% of patients, underscoring the pivotal role of tumor control in disease remission (9).

In contrast, the coexistence of myasthenia gravis (MG) with CD is far less common. The overlap is thought to involve IL-6–driven immune activation, B-cell hyperactivity, and autoantibody production (10). Reported cases suggest a younger patient predominance and a tendency for tumors to localize in the mediastinum (11). Although MG and PNP have each been independently associated with CD, their simultaneous occurrence is rare. To date, only four such cases have been reported, and this case represents a rare fifth occurrence involving the mixed-type histopathological variant of UCD, further expanding the known clinicopathologic spectrum. The triad of CD, MG, and PNP therefore represents a highly unusual coexistence of mucocutaneous, neuromuscular, and lymphoproliferative disorders. Such cases pose substantial diagnostic and therapeutic challenges, requiring close collaboration among thoracic surgery, neurology, dermatology, pathology, and immunology. This case thus expands the immunopathologic spectrum of CD and underscores the importance of multidisciplinary management in such rare and complex cases.

## Case presentation

**Figure 1:**
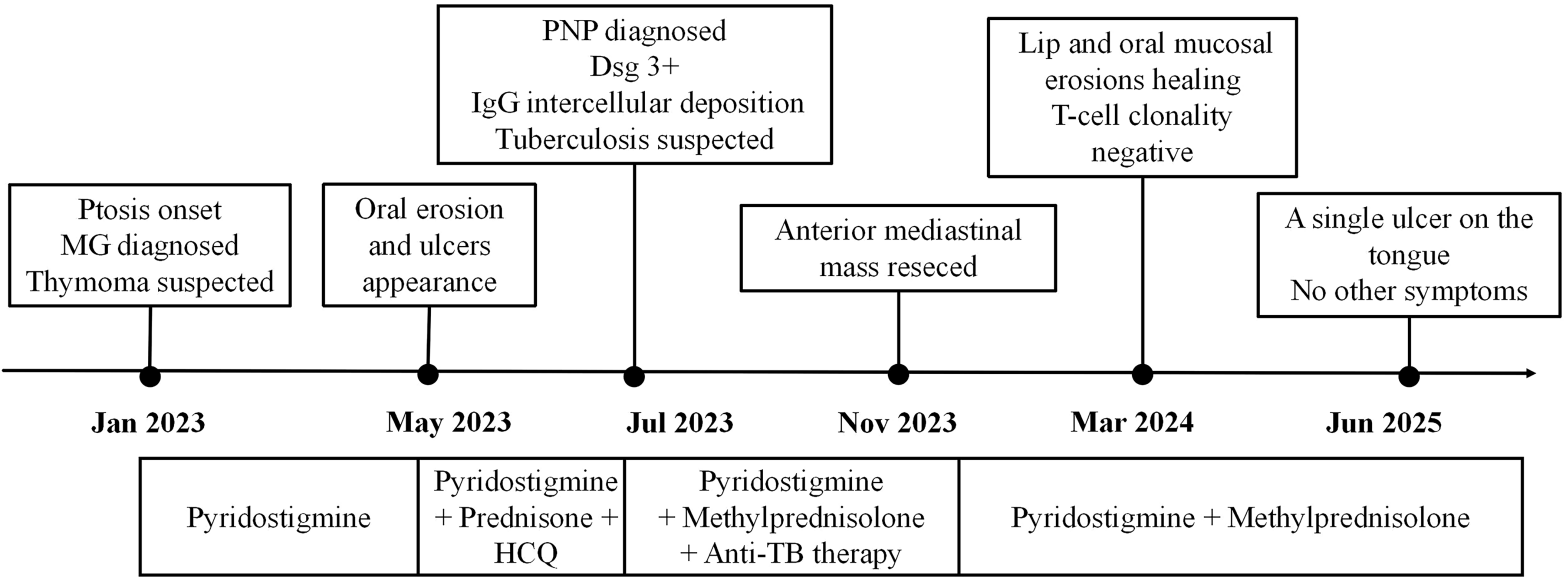
Summary of the diagnostic and therapeutic process in a patient with unicentric Castleman disease complicated by myasthenia gravis and paraneoplastic pemphigus.

**Figure 2:**
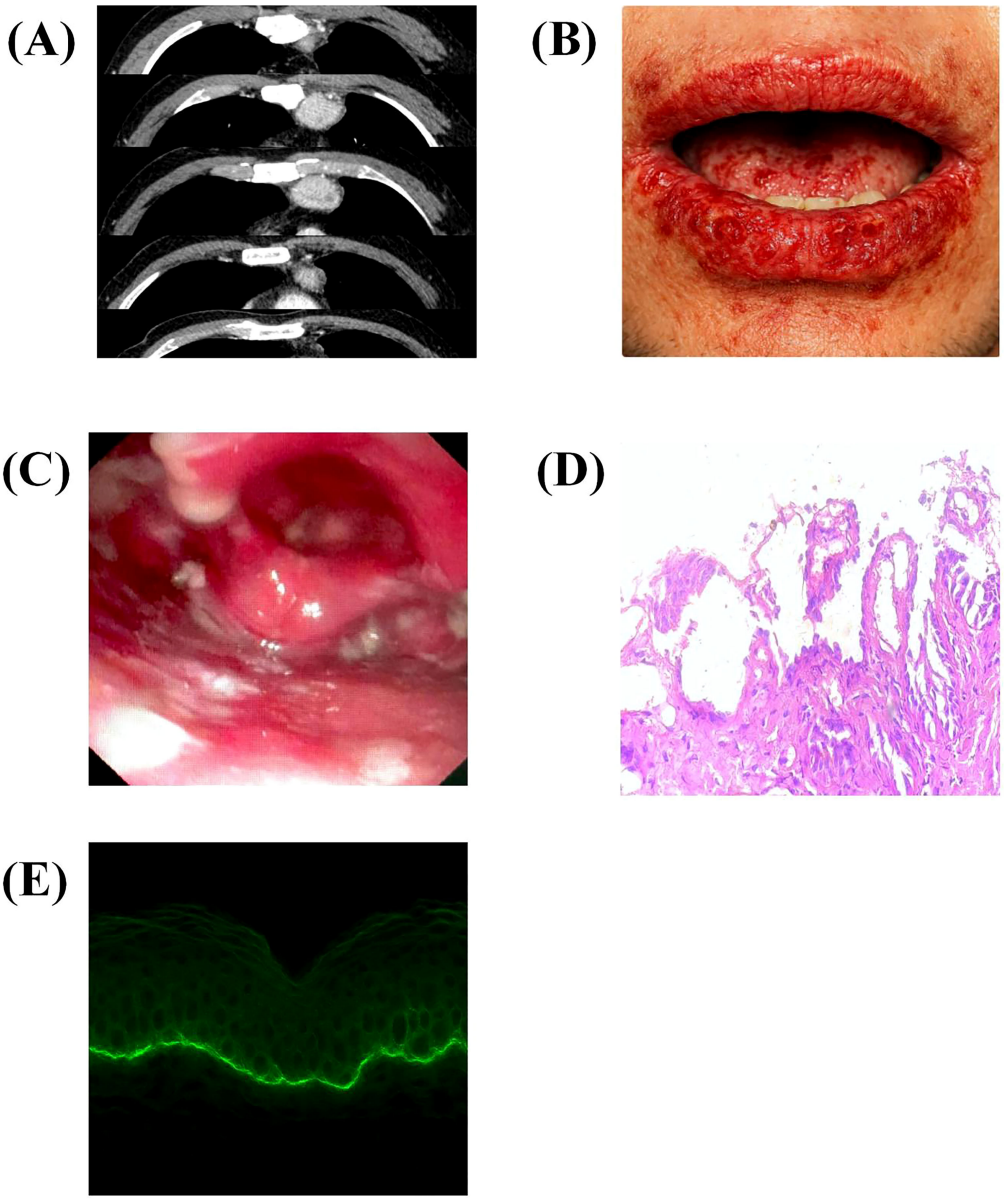
Clinical, radiological, and pathological findings of the patient. **(A)** Chest CT scan showing a retrosternal soft tissue mass measuring approximately 41 × 32 mm, located in the anterior mediastinum. **(B)** Pretreatment photograph showing hemorrhagic crusts and erosions of the lips (arrow). **(C)** Pretreatment endoscopic image demonstrating ulcerations involving the pharynx and epiglottis (arrow). **(D)** Hematoxylin and eosin staining revealing suprabasal acantholysis (arrow). **(E)** Direct immunofluorescence showing strong linear IgG deposition along the basement membrane zone with minimal intercellular staining.

In January 2023, a 49-year-old man presented with a two-month history of right upper eyelid ptosis, characterized by diurnal fluctuation and worsening after exertion (**Figure 1**). He had no relevant family or past medical history. Neurological examination and a positive neostigmine test supported the diagnosis of ocular myasthenia gravis (MG). Laboratory findings showed positive anti-PM-Scl antibodies and antinuclear antibody (ANA, 1:80, nuclear granular pattern). In contrast, C-reactive protein (CRP), erythrocyte sedimentation rate, anti-neutrophil cytoplasmic antibodies, immunoglobulin G, and HIV were within normal limits. Serologic testing for anti–acetylcholine receptor and anti–MuSK antibodies was not performed because the diagnosis of myasthenia gravis was already well established. Symptomatic treatment with pyridostigmine (60 mg, three times daily) resulted in significant clinical improvement. During the diagnostic work-up, chest imaging revealed a well-defined anterior mediastinal mass, initially considered suspicious for thymoma (**Figure 2A**).

In May 2023, the patient developed progressive oral and pharyngeal erosions and ulcers as shown in **Figures 2B, C**. Evaluation by stomatology and dermatology confirmed extensive mucosal involvement. The patient was clinically suspected to have a bullous autoimmune disorder and empirical immunosuppressive therapy with hydroxychloroquine and oral prednisone showed minimal benefit. Histopathological examination of mucosal biopsies revealed suprabasal acantholysis (**Figure 2D**). Serologic testing performed at an outside facility demonstrated positivity for envoplakin and periplakin antibodies, further supporting the diagnosis of paraneoplastic pemphigus, a hallmark feature supporting PNP diagnosis. Direct immunofluorescence confirmed strong linear IgG deposition along the basement membrane zone (**Figure 2E**), while ELISA showed significantly elevated anti-desmoglein-3 antibody levels (143.5 U/mL). Collectively, these findings provided robust evidence distinguishing PNP from other blistering disorders and underscored the importance of combining histology and immunopathology for definitive diagnosis. The interferon-γ Release Assay was positive and prophylactic isoniazid combined with rifampin was initiated for latent tuberculosis infection. Systemic corticosteroids (methylprednisolone 40 mg IV daily and oral 12 mg/day) and supportive therapy provided partial symptom control under multidisciplinary care involving neurology and dermatology.

**Figure 3:**
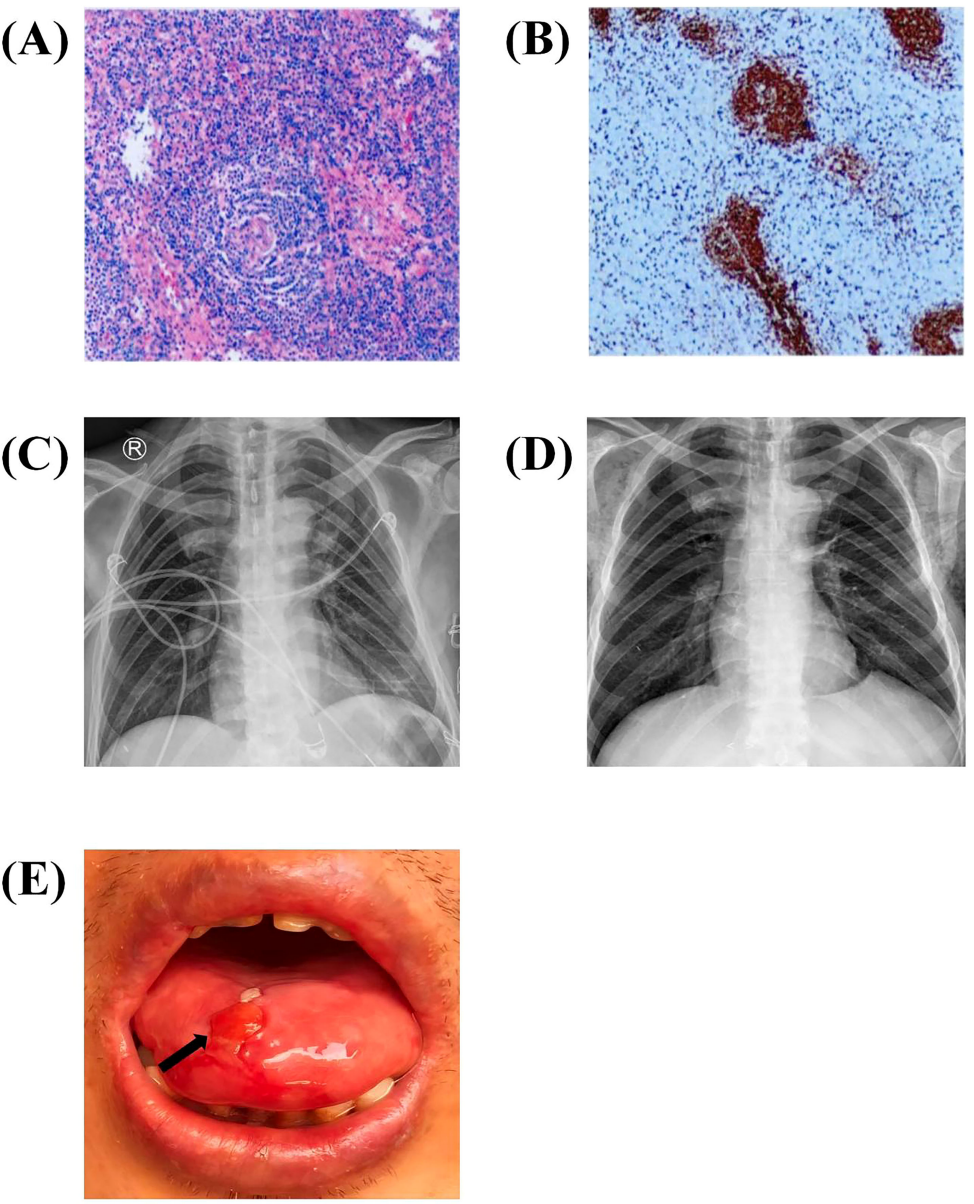
Histopathological and immunohistochemical features, postoperative imaging and follow-up findings. **(A)** Hematoxylin and eosin staining demonstrating lymphoid follicular hyperplasia with regressed germinal centers and expanded mantle zones forming concentric “onion-skin”–like layers. **(B)** CD20 immunohistochemistry highlighting well-defined B-cell follicles. **(C)** Chest radiograph obtained two days after surgery, showing well-expanded lungs without pneumothorax. **(D)** Chest radiograph obtained two weeks after surgery, demonstrating good recovery with clear lung fields and no pleural effusion. **(E)** Follow-up photograph in June 2025 showing marked improvement of mucocutaneous lesions on the lips and tongue (arrow).

Once neuromuscular and mucocutaneous symptoms were stabilized, the patient underwent surgical resection of the anterior mediastinal mass in November 2023. Histopathological examination confirmed UCD of mixed type (**Figures 3A, B**). Postoperative recovery was uneventful (**Figures 3C, D**), with elevated serum IL-6 levels (12 pg/mL). To date, only four previous cases of this subtype complicated by both MG and PNP has been reported.

At follow-up in March 2024, the patient showed significant clinical improvement. Oral lesions had nearly resolved under maintenance oral methylprednisolone therapy (16 mg/day), with only a small residual tongue ulcer (**Figure 3E**), and no recurrence of myasthenic symptoms was observed, validating the effectiveness of combined surgical and immunosuppressive therapy. T-cell receptor gene rearrangement was negative, excluding angioimmunoblastic T-cell lymphoma. The patient exhibited excellent adherence to the prescribed therapeutic regimen, with no treatment-related adverse events or unanticipated complications documented during the clinical course. During follow-up in June 2025, the patient reported substantial improvement in quality of life, expressing relief after the definitive diagnosis and the resolution of mucocutaneous and neuromuscular symptoms. From the patient’s perspective: “At first, I felt very anxious because the diagnosis was uncertain, and the painful mouth sores made eating and speaking difficult. After the surgery and continued treatment, my symptoms gradually improved, and I was relieved to finally understand my condition. I am deeply grateful to the doctors from thoracic surgery, dermatology, and neurology for working together. I hope that sharing my experience can help other patients with similar rare diseases receive timely diagnosis and coordinated care”.

## Discussion

CD is a rare lymphoproliferative disorder that has been increasingly associated with autoimmune conditions, particularly UCD. Although PNP and MG have each been independently reported in association with CD, their simultaneous occurrence is exceedingly rare. The present case represents mixed-type UCD complicated by both MG and PNP, a constellation of disorders that has been reported only in a few cases in the literature (**Table 1**). Our findings are consistent with recent reviews and systematic analyses showing that PNP/paraneoplastic autoimmune multiorgan syndrome typically arises in the context of lymphoproliferative disorders, with Castleman disease representing a major associated entity (7). Furthermore, scoping reviews focusing on oral PNP emphasize the predominance of severe erosive stomatitis and the diagnostic relevance of envoplakin and periplakin antibodies, which were also detected in our patient (12). This case initially posed a diagnostic challenge, as the anterior mediastinal mass closely resembled a thymic epithelial tumor on imaging, a frequent differential diagnosis in patients with myasthenia gravis. The isolated anti–PM-Scl positivity likely reflected generalized B-cell activation rather than a specific overlap syndrome, consistent with cytokine-driven autoimmunity reported in CD.

**Table 1:** Reported cases of Castleman disease with concurrent paraneoplastic pemphigus and myasthenia gravis, 1990–2025.

Study	Year	Gender	Age, years	Mucosal involvements	CD type	Autoantibodies	Tumour location	Histological type	Myasthenic crisis
Chorzelski T et al. (13)	1999	M	31	oral erosions	UCD	Anti-plakin positive	Anterior mediastinal	Hyaline vascular	No
Jakubíková M et al. (14)	2013	M	31	oral and penis mucositis	UCD	Anti-Dsg3+, ANA–	Retroperitoneal	Mixed	Yes
Dinesha et al. (15)	2014	F	25	oral mucositis	UCD	Dsg3+	Retroperitoneal	Hyaline vascular	No
Barry KK et al. (16)	2022	F	7	oral mucositis	UCD	anti-plakin not tested	Retroperitoneal	Unknown	No
Present case	2023	M	49	oral mucositis	UCD	Envoplakin+/Periplakin+, Dsg3+, Dsg1–	Anterior mediastinal	Mixed	No

We reviewed the case reports of CD complicated by MG and PNP reported in the literature over the past 35 years (13–16). Only four concurrent cases of CD have been reported. Among these, one case (20%) involved a child, three cases (60%) occurred in young adults, and one case (20%) in a middle-aged adult, supporting the observation that this triad predominantly affects younger individuals. To date, five patients have been reported, including the present case: two with mixed-type CD, two with hyaline vascular CD, and one in which the histological subtype was not specified. The present case underscores that the hyaline vascular and mixed type may both serve as substrates for aberrant immune activation and complex autoimmune comorbidities. All five reported cases underwent symptomatic treatment to stabilize their conditions before surgical resection of the lesion, and one patient experienced a postoperative myasthenic crisis.

In this case, the anterior mediastinal mass was initially suspected to be a thymoma, which is a common association with MG. Lymphoma and other lymphoproliferative disorders were also considered in the differential diagnosis. Only histopathological examination confirmed the diagnosis of mixed-type UCD, underscoring the importance of tissue diagnosis in such complex presentations.

Currently, there is no definitive evidence supporting a direct causal link between PNP and MG in CD. Reported cases have shown variable sequences of onset for MG and PNP, suggesting that the two conditions may be driven by distinct immunopathogenic mechanisms. Notably, among previously reported cases of the CD–MG–PNP triad, one patient succumbed to sepsis with multi-organ failure following surgery due to incomplete resection of a tumor tightly adherent to major vessels (14), further emphasizing the critical importance of complete surgical excision in controlling immune activation (17).

**Figure 4:**
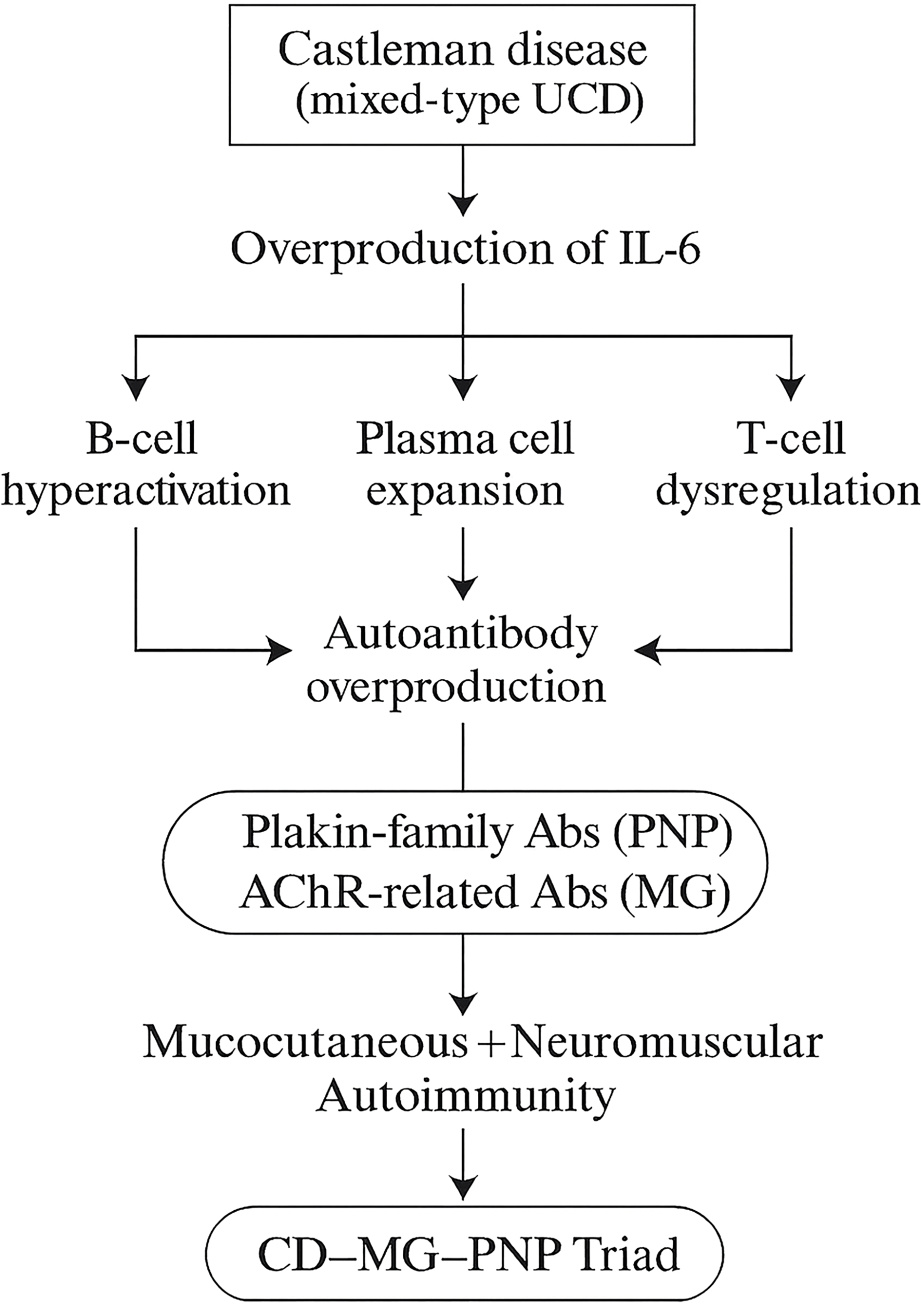
IL-6–driven immunopathologic mechanism linking Castleman disease, paraneoplastic pemphigus, and myasthenia gravis. In mixed-type unicentric CD, excess IL-6 drives B-cell hyperactivation, plasma-cell expansion, and T-cell dysregulation, resulting in overproduction of plakin-family antibodies (PNP) and acetylcholine-receptor–related antibodies (MG). These combined autoimmune processes lead to the clinical CD–MG–PNP triad.

From an immunopathological perspective, mixed-type UCD is particularly relevant to interleukin-6 (IL-6)–driven immune dysregulation. IL-6 is a well-established driver of B-cell hyperactivation and autoantibody production, processes central to the pathogenesis of both MG and PNP (18, 19). Elevated serum IL-6 levels correlate with disease activity in AChR-positive MG patients (20). In our patient, serum IL-6 levels were modestly elevated (12 pg/mL), which paralleled the coexistence of both neuromuscular weakness and mucocutaneous lesions. Moreover, the characteristic suprabasal acantholysis and IgG deposition along the basement membrane zone illustrated in **Figures 2D, E** not only confirmed the diagnosis of PNP but also highlighted the central role of autoantibody-mediated epithelial damage in the disease pathogenesis. In experimental autoimmune MG models, IL-6 receptor blockade ameliorates muscle weakness and reduces both the production and deposition of AChR autoantibodies (21). Persistent IL-6 overexpression within the hyperplastic plasma cell–rich microenvironment may facilitate excessive antibody generation, including anti–acetylcholine receptor antibodies and anti–desmoglein-3 antibodies, thereby linking CD directly to neuromuscular and mucocutaneous autoimmunity (22), as shown in **Figure 4**. Moreover, the abnormal lymphoid follicles and hypervascular stroma of Castleman lesions may expose tumor-associated self-antigens, further amplifying autoimmune cross-reactivity (18).

Given the central role of IL-6 in immune dysregulation, it is not surprising that targeted therapies against IL-6 have demonstrated clinical efficacy. In multicentric cases, agents such as siltuximab and tocilizumab have shown efficacy in controlling inflammation and reducing lymphadenopathy. Although evidence is limited in unicentric disease, their mechanism suggests potential value for patients with complicated autoimmune manifestations, particularly when surgery is not feasible or symptoms persist despite resection and immunosuppression. Further studies are needed to clarify their role in such rare scenarios (23).

The clinical management of this patient highlights the importance of multidisciplinary care. Initial stabilization of MG with anticholinesterase therapy and subsequent immunosuppressive treatment for PNP provided a foundation that allowed safe surgical resection of the mediastinal mass. Complete surgical excision, combined with tailored immunosuppressive therapy, was essential for durable control of symptoms and prevention of postoperative myasthenic crisis. This sequence of neurology, dermatology, pathology, and thoracic surgery input underscores the necessity of coordinated, multidisciplinary strategies in complex cases.

In summary, this case describes a rare occurrence of mixed-type UCD complicated by both MG and PNP, with four previous report in the literature. The diagnostic process was complex, requiring integration of multiple tests and close collaboration across specialties. Multidisciplinary management proved essential for accurate diagnosis, safe surgery, and effective long-term treatment. Further case accumulation is needed to improve understanding and guide management of this rare disease constellation.

This report has several limitations. It presents a single rare case, which precludes establishing a causal relationship among Castleman disease, myasthenia gravis, and paraneoplastic pemphigus. In addition, although the follow-up period exceeded 20 months, it may still be insufficient to evaluate long-term recurrence or immunological evolution and the lack of longitudinal follow-up of autoantibody titers after resection of the CD lesion. Further case accumulation and longer follow-up are needed to validate these findings and elucidate the underlying mechanisms.

## Data Availability

The original contributions presented in the study are included in the article/supplementary material. Further inquiries can be directed to the corresponding authors.
